# Optimization of Extraction Process and Analysis of Biological Activity of Flavonoids from Leaves of Cultivated ‘Qi-Nan’ Agarwood

**DOI:** 10.3390/molecules29081828

**Published:** 2024-04-17

**Authors:** Qingle Li, Penglian Wei, Yingjian Li, Yunlin Fu

**Affiliations:** School of Forestry, Guangxi University, Nanning 530011, China; 2109302009@st.gxu.edu.cn (Q.L.); weipenglian@gxu.edu.cn (P.W.); liyingjian@gxu.edu.cn (Y.L.)

**Keywords:** agarwood, ‘Qi-Nan’ leaves, flavonoid, identification of ingredients, extraction, biological activity

## Abstract

Currently, the planting of ‘Qi-Nan’ is continuously increasing, yet a substantial amount of ‘Qi-Nan’ leaves have not been properly exploited. To improve the ‘Qi-Nan’ tree ’s utilization value, ‘Qi-Nan’ leaves were used as a raw material. An ultrasound-assisted method was performed to obtain the flavonoids from the ’Qi-Nan’ leaves, followed by optimization of the extraction factors using a one-way and response surface methodology to enhance the extraction of flavonoids. Subsequently, the composition of the flavonoids, as well as their bioactive abilities, were analyzed by ultra-high-performance liquid chromatography–mass spectrometry (UHPLC-MS) and in vitro activity testing methods. The findings demonstrated that a 1:50 material-to-liquid ratio, 60% ethanol concentration, and ultrasound-assisted extraction time of 30 min were the ideal procedures for extracting flavonoids (flavonoid content: 6.68%). Meanwhile, the ‘Qi-Nan’ leaves possessed the antioxidant and medicinal potential to prevent diabetes and Alzheimer ’s disease, as evidenced by the semi-inhibitory concentrations (IC50 values) of flavonoid extracts for scavenging DPPH^•^ free radicals, scavenging ABTS^•+^ free radicals, inhibiting acetylcholinesterase, and inhibiting α-glucosidase, which were 12.64 μg/mL, 66.58 μg/mL, 102.31 μg/mL, and 38.76 μg/mL, respectively, which indicated that the ‘Qi-Nan’ leaves possessed the properties of antioxidant and medicinal potential for the prevention of Alzheimer ’s disease and diabetes.

## 1. Introduction

Agarwood, a resinous heartwood that belongs to the *Aquilaria* species (Thymelaeaceae) [1], is regularly used in aromatherapy, medicine, the fragrance industry, religion, and other fields. The 21 species in the *Aquilaria* are mainly found in approximately 20 countries along the India–Malaysia–Papua New Guinea route, among which *Aquilaria sinensis* is mainly distributed in China. Agarwood is particularly valuable, and the best quality and most valuable kind is wild ‘Qi-Nan’ agarwood, which is also extremely rare [2,3]. In recent years, the rapid development of high-quality seed breeding and cultivation techniques has resulted in the emergence of grafted Aquilaria sinensis (namely, ‘Qi-Nan’). It has developed quickly in China owing to its greater agarwood output and similar quality to wild ‘Qi-Nan’ agarwood [4]. Unfortunately, the leaves of the ‘Qi-Nan’ are frequently thrown away during the harvesting of valuable agarwood, resulting in a waste of resources. 

It has been reported that *Aquilaria* leaves contain a variety of compounds such as flavonoids, polysaccharides, amino acids, and phenols [5,6,7,8]. Among them, flavonoids such as genkwanin, luteolin [9], suberone, and Hydroxygenkwanin are the most predominant active components of the *Aquilaria* leaves. It is worth recognizing that flavonoids have been found to be antioxidants [10,11,12], hypoglycaemic [12,13,14], and anti-Alzheimer’s disease [13].

Within the intricate processes of the human metabolism, an abundant onslaught of free radicals is invariably produced. Should there be an overabundance, these reactive entities wreak havoc upon cellular integrity, precipitating the onset of maladies such as hypertension, cardiovascular and cerebrovascular afflictions, and diabetes mellitus, among others. In addition, acetylcholinesterase and a-glucosidase are key targets for the treatment of Alzheimer’s disease and diabetes mellitus, but clinically useful drugs such as acarbose, tacrine, and voglibose are expensive and have serious side effects. As a result, natural active ingredients from plants—flavonoids—are receiving increasing attention. The extraction methods for flavonoids primarily include the ethanol reflux method, ultrasonic extraction method, enzyme extraction method, and microwave extraction method. Rightfully, the ultrasonic-assisted extraction method is widely used in the extraction of plant flavonoids by accelerating the destruction of the cell wall or cell membrane of the plant cells using ultrasonic waves to induce the intracellular flavonoids to be dissolved in the extraction solution, which has advantages such as simple operation, a short extraction time, and a high efficiency of extraction [15,16].

However, most current studies have focused on the leaves of *Aquilaria sinensis* (Lour.), while few studies have been conducted on the identification, extraction, and bioactivity of flavonoids in the ‘Qi-Nan’ leaves. Based on that, in this study, the flavonoids in the leaves of ‘Qi-Nan’ were extracted, and the extraction procedure of flavonoids was optimized. Then, the flavonoids in ‘Qi-Nan’ leaves were characterized by ultra-high performance liquid chromatography–mass spectrometry (UHPLC-MS), and their antioxidant, acetylcholinesterase inhibition, and α-glucosidase inhibition activities were determined. The results will provide a theoretical basis for the development and utilization of ’Qi-Nan’ leaves and a potential source of raw materials for the food and pharmaceutical industries.

## 2. Results and Analyses

### 2.1. UHPLC-MS

**Figure 1:**
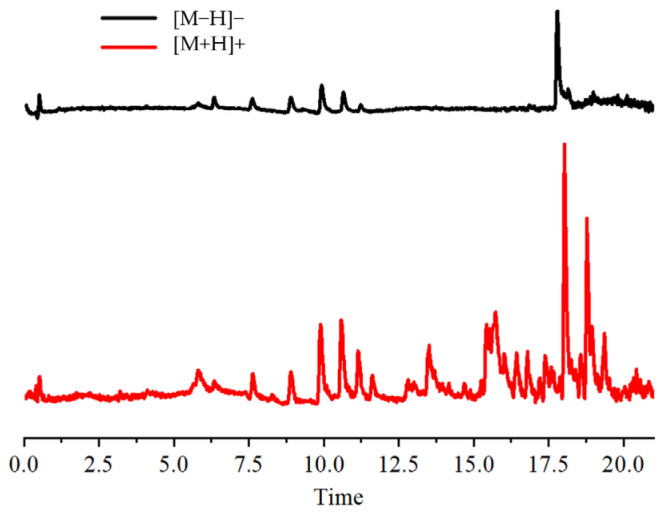
Total ion chromatogram.

The total ion flow chromatograms for [M+H]^+^ and [M−H]^−^ are displayed in Figure 1. The flavonoids in the leaves of ‘Qi-Nan’ were analyzed using the UHPLC-MS procedure. Based on the chemical constituents’ high-resolution mass spectrometry (HRMS) data, retention times, mass spectrometry databases, and references to primary and secondary mass spectrometry information for the flavonoids (Table 1), a total of 10 flavonoids were identified. These were Quercetin, Vicenin-2, Cynaroside, Miquelianin, Glycitin, Luteolin, Sakurantein, Hispidulin, Glycitein, and Scrophulein. According to reports [17,18,19,20,21,22,23], flavonoids, including glucoside, wogonin, sakuranin, and kaempferol-7-O-glucoside, have antioxidant, antibacterial, glucosidase inhibition, and acetylcholinesterase inhibition abilities, which can provide a basis for subsequent research.

**Figure 2:**
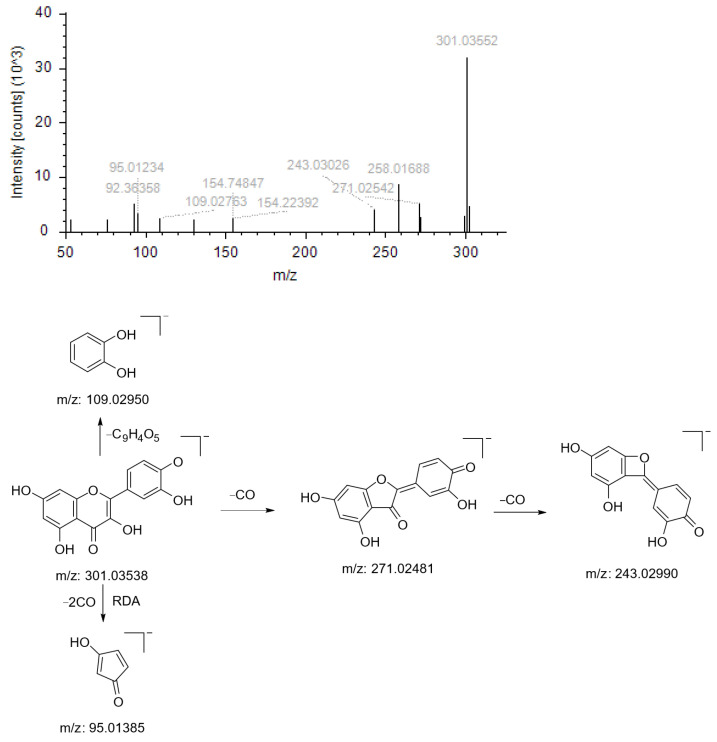
Ion fragmentation maps and cleavage path maps of Compound NO.1.

Compound NO.1’s quasimolecular ion peak *m*/*z* 301.03552 [M+H]^+^ has the chemical formula C_15_H_10_O_7_; this parent ion (*m*/*z* 301.03552) first loses one molecule of CO to produce the fragmentation ion *m*/*z* 271.02542, followed by the loss of another molecule of CO to produce the fragmentation ion m/z 243.03026; this parent ion (*m*/*z* 301.03552) undergoes RDA cleavage and loses two molecules of CO to produce the fragmentation ion *m*/*z* 95.01234; the parent ion (*m*/*z* 301.03552) undergoes a simple rupture to generate the fragment ion *m*/*z* 109.02763. Based on the literature and the mass spectrometry library, the compound is presumed to be Quercetin (Figure 2).

**Figure 3:**
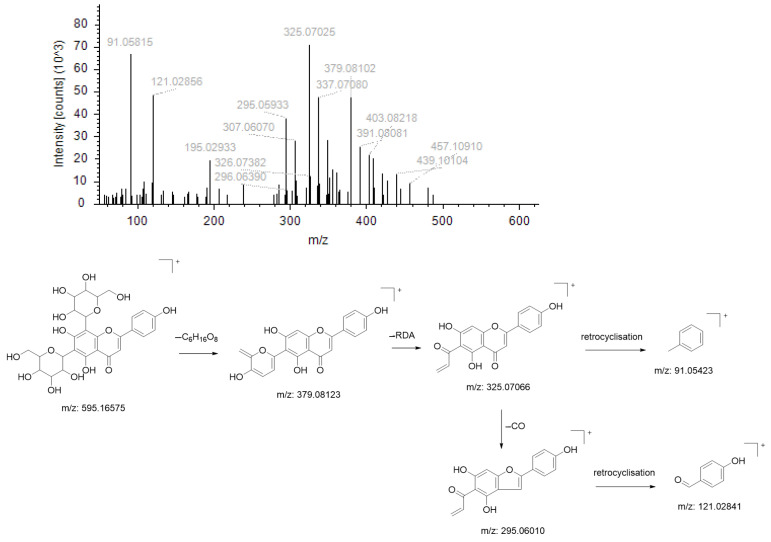
Ion fragmentation maps and cleavage path maps of Compound NO.2.

Compound NO. 2’s quasimolecular ion peak *m*/*z* 595.16575 [M+H]^+^ has the chemical formula C_27_H_30_O_15_, and this parent ion (*m*/*z* 595.16575) firstly dehydrogenates one molecule of sugar and three molecules of water to produce the fragment ion *m*/*z* 379.08102; that fragment ion then undergoes RDA cleavage to produce the fragment ion *m*/*z* 325.07025, which finally undergoes retrocyclisation and dehydration to produce the fragment ion *m*/*z* 91.05815; this parent ion (*m*/*z* 325.07025) loses one molecule of CO to produce the fragment ion *m*/*z* 295.05933, followed by reverse cyclisation to produce the fragment ion *m*/*z* 121.02856. Based on the literature and the mass spectrometry library, the compound is presumed to be Vicenin-2 (Figure 3).

The ion fragmentation maps and cleavage path maps of the other compounds are presented in the Supplementary Material.

### 2.2. Results and Analyses of Single-Factor Experiments

#### 2.2.1. Ethanol Concentration

**Figure 4:**
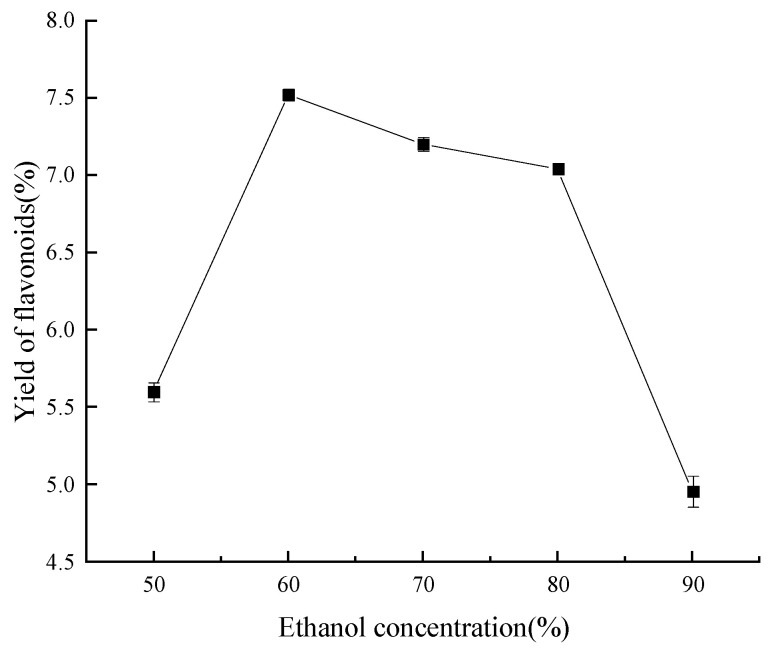
Effect of volume fraction of ethanol on yield of flavonoids.

As seen in Figure 4, the maximum flavonoid extraction rate was achieved at a concentration of 60% ethanol. Specifically, once the ethanol concentration was below 60%, the rate of flavonoid extraction rose as the ethanol concentration increased; nevertheless, when the ethanol concentration was above 60%, the reverse phenomenon was noted. This phenomenon may be explained by the fact that different ethanol concentrations have different polarities; as the ethanol concentration increases, so does the ethanol’s capacity to break hydrogen bonds and hydrophobic interactions between flavonoids and proteins, polysaccharides, and other substances. Hence, more flavonoids dissolve, and the yield is increased. It is worth noting that when the concentration of ethanol is excessive, the polarity difference between the flavonoid compounds in ‘Qi-Nan’ leaves and the solvent increases. This discrepancy contributes to a diminished solubility of the flavonoids. Furthermore, certain impurities that are soluble in alcohol, along with pigments and lipophilic substances, exhibit enhanced solvation. These entities compete with the flavonoids for interactions with the ethanol–water molecules, culminating in a reduced recovery rate of flavonoids [33]. Consequently, an ethanol concentration of 60% was adjudicated to be optimal for subsequent refinement trials.

#### 2.2.2. Material–Liquid Ratio

**Figure 5:**
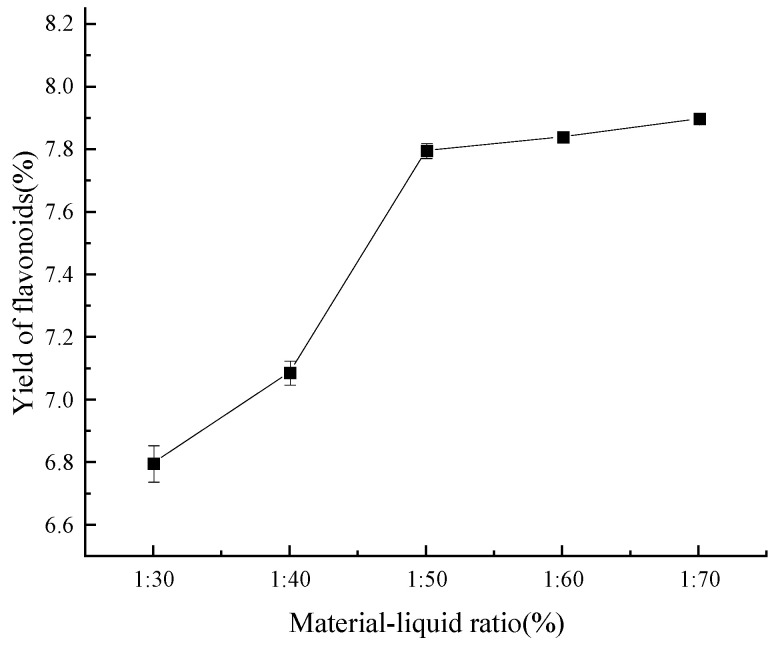
Effect of material–liquid ratio on yield of flavonoids.

As is evident from Figure 5, when the solid–liquid ratio increased, the extraction rate of flavonoids progressively increased. On the other hand, the flavonoid extraction rate achieved its critical value at a solid–liquid ratio of 1:50 (g/mL), indicating that the extraction rate remained constant at higher ratios. This phenomenon is most likely caused by the fact that, at a certain ratio, all flavonoids dissolve in ethanol, while an insufficient amount of ethanol does not dissolve any flavonoids at all [34]. Therefore, a material–liquid ratio of 1:50 (g/mL) was chosen for further optimization based on the principle of the most economical of the testing process.

#### 2.2.3. Extraction Time

**Figure 6:**
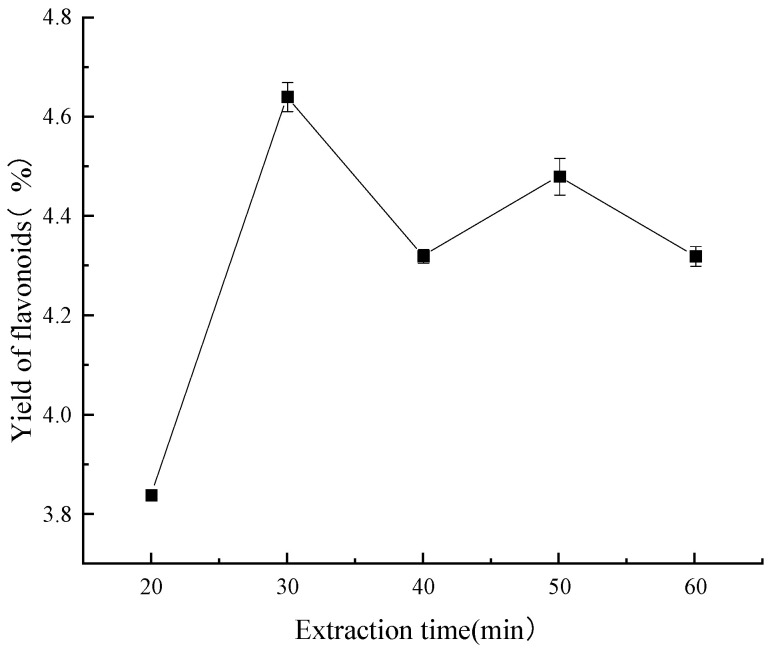
Effect of extraction time on yield of flavonoids.

As depicted in Figure 6, after 20 min, it was found that the ultrasound-assisted extraction time was still too short, which resulted in partially solubilized flavonoids in the sample and a lower-than-ideal extraction rate of these compounds. When the flavonoid extraction process was advanced to a 30 min interval, it seemed to reach a basic condition of equilibrium and produce the highest extraction rate. Reference [35] indicates that abnormalities resulting from contaminants within the specimen could be the cause of the observed drop at the 40 min mark. If the process was continued for more than fifty minutes, the number of flavonoids decreased. This was probably due to an extraction period that was too long, which would have damaged the integrity of the thermolabile components. 

### 2.3. Analysis of Response Surface Optimization Extraction Methods

#### 2.3.1. Box–Behnken Experimental Design and Results

According to the principle of a Box–Behnken experimental design (BBD), in Design-expert 11 software, a three-factor, three-level experiment was designed with the material–liquid ratio, ultrasound-assisted extraction time, and ethanol concentration as variables, and the extraction rate of flavonoids from the leaves of ‘Qi-Nan’ as the only response value, respectively. The results were as follows (Table 2, Table 3 and Table 4).

The quadratic multinomial regression equations for the flavonoid extraction rate (*Y*) of ‘Qi-Nan’ leaves based on the material–liquid ratio (*A*), ethanol concentration (*B*), and ultrasound-assisted extraction time (*C*) were as follows:

$$Y=7.10+0.0031A+0.1867B\;-\;0.0308C\;-\;0.0186AB\;-\;0.0656AC+0.1440BC\;-\;0.5462A^{2}\;-\;0.5258B^{2}-\;0.2833C^{2}$$

The results clearly demonstrated that the developed model was significant, with a very small *p* value (<0.05). The F value of the lack of fit was calculated to be 0.7907, with a nonsignificant difference in variance. In particular, the gap between the adjusted R^2^ and the predicted R^2^ was less than 0.2, and the Adeq precision value was greater than 4, which demonstrated the remarkable proximity of the developed model for forecasting the variations. Correspondingly, the effects of the three factors on the extraction rate were ethanol concentration > ultrasound-assisted extraction time > material–liquid ratio.

#### 2.3.2. Response Surface Analysis (RSA)

**Figure 7:**
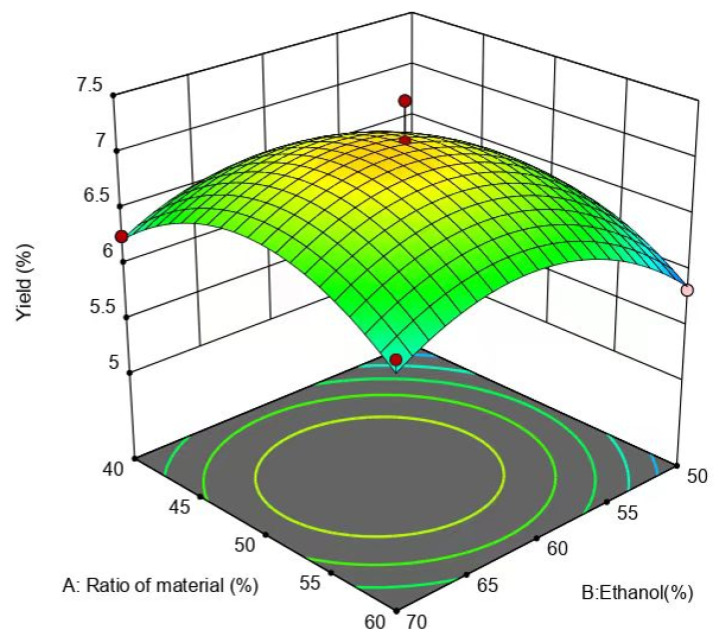
Effect of ethanol concentration and liquid-to-ethanol ratio on yield of flavonoids.

From Figure 7, it can be seen that the slope of the response surface is steeper and the contours are circular, but the surfaces have obvious color differences, indicating that the interaction between the ethanol concentration and feed ratio is not high, and the surface of the ethanol concentration is steeper relative to the surface of the feed ratio, suggesting that the effect of the ethanol concentration on the yield of flavonoids is greater than that of the feed ratio.

**Figure 8:**
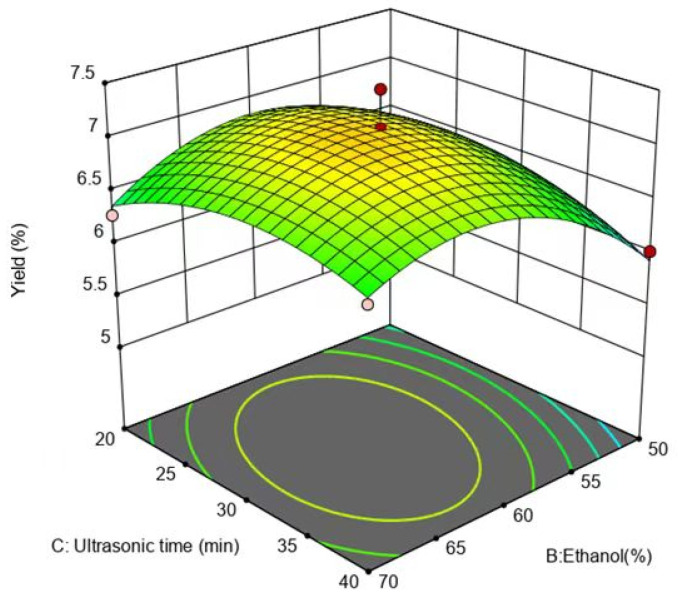
Effect of ethanol concentration and extraction time on yield of flavonoids.

Figure 8 illustrates that the slope of the response surface was steeper, and the contours were elliptical, indicating that the interaction between the ethanol concentration and ultrasound-assisted extraction time was more powerful. Intriguingly, the response surface for the ethanol concentration was steeper relative to that of the ultrasound-assisted extraction time, demonstrating that the impact of the ethanol concentration on the flavonoid yield was more important than the effect of the ultrasound-assisted extraction time.

**Figure 9:**
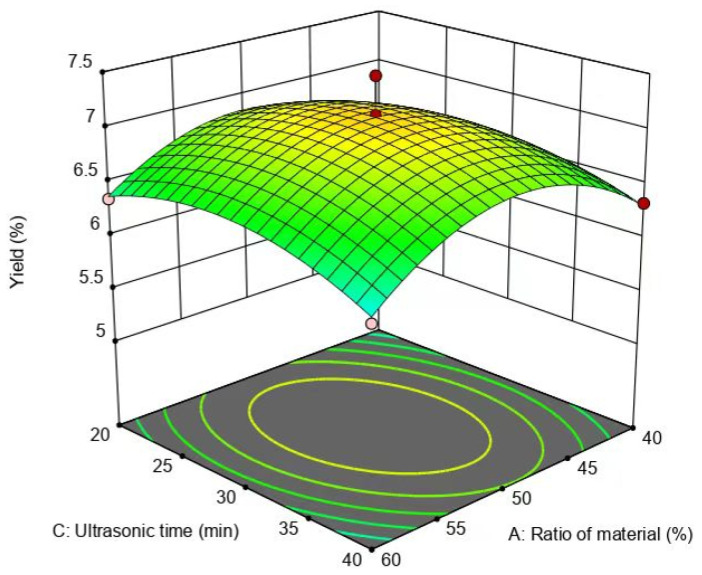
Effect of material–liquid ratio and extraction time on the yield of flavonoids.

The interaction of the material–liquid ratio and ultrasound-assisted extraction time exhibited a negligible effect on the flavonoid yield, because the slopes of the response surfaces were the flattest (Figure 9). However, the surface of the ultrasound-assisted extraction time was steeper compared to the surface of the material–liquid ratio (Figure 9), pointing out that the impact of ultrasound-assisted extraction time on the yield of flavonoids was marginally greater than that of the material–liquid ratio.

#### 2.3.3. Optimization of the Extraction Process

Based on the analysis of the selected model, the optimum process for flavonoid extraction recommended in the Design-expert 11 software to obtain the maximum ‘Qi-Nan’ flavonoids yield was as follows: a material–liquid ratio of 1:50.0032, ethanol concentration of 61.7578%, and ultrasonication time of 29.9 min. Considering the operational problems, it was determined that the optimum process was a material–liquid ratio of 1:50, ethanol concentration of 60 %, and ultrasonication time of 30 min. Under these conditions, three parallel experiments were carried out, and the average yield of flavonoids was 6.68%, which was nearly the theoretical value (6.71%). Such a yield was remarkably superior to the yields of 2.88%, 3.10%, and 5.62% obtained from *Aquilaria sinensis* leaves by Duan et al. [33], Li et al. [36], and Su et al. [37], respectively, presumably due to different germplasms leading to different results. In addition, the yield was also substantially higher compared to the 3.78% yield of flavonoids extracted from ‘Qi-Nan’ leaves by the ethanol reflux method by Lin et al. [38]; such a difference was caused by the extraction methods. In summary, the flavonoid content in the leaves of ‘Qi-Nan’ was more abundant, and the optimized extraction was performed more effectively.

### 2.4. Antioxidant Capacity

**Figure 10:**
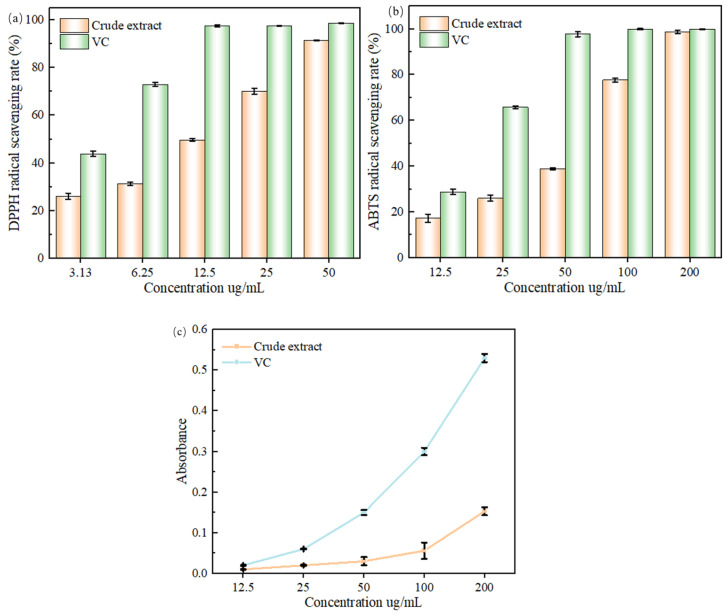
(**a**) Effect of flavonoid extract and DPPH^•^ mass concentration on scavenging rate of DPPH^•^ radicals. (**b**) Effect of flavonoid extract and VC mass concentration on ABTS^•+^ radical scavenging rate. (**c**) Effect of different concentration samples on total reducing power.

As illustrated in Figure 10a, the inhibition of acetylcholinesterase activity increased and then leveled off, exhibiting an obvious dose dependence. The positive control tacrine inhibited acetylcholinesterase activity more effectively than the extract in the range of 31.3–1000 µg/mL. However, once the concentration of the extract solution was 1000 µg/mL, the inhibition of acetylcholinesterase activity reached 99.18 ± 0.76 %, which was comparable to that of tacrine (100 ± 0.73 %). The IC_50_ values of the extract and tacrine were 102.31 ± 2.43 and 0.08 ± 0.03 μg/mL, respectively suggesting that the flavonoid extracts of the leaves of ‘Qi-Nan’ possessed the ability to inhibit acetylcholinesterase activity.

Figure 10b effect of flavonoid extract and VC mass concentration on ABTS^•+^ radical scavenging rate. Additionally, over a certain concentration range, the thatch berry root flavonoid extract’s capacity to inhibit acetylcholinesterase was positively connected with its mass concentration, with an IC50 value of 400.3 μg/mL, which was comparable to the findings of the current investigation.

It is generally accepted that an IC50 value of less than 10 mg/mL for a substance indicates that the substance has good antioxidant properties [39], and the results showed that the flavonoid extracts of ‘Qi-Nan’ leaves had a strong DPPH^•^ radical scavenging ability and a strong ABTS^•+^ radical scavenging ability. The ‘Qi-Nan’ leaves flavonoid extract has a stronger DPPH^•^ radical scavenging ability than the agarwood leaves flavonoid extract extracted by Duan et al. [33]. The ‘Qi-Nan’ leaves flavonoid extract had a stronger ABTS^•+^ radical scavenging ability than the Perilla frutescens leaves flavonoid extract extracted by Yi et al. [40]. Both radical scavenging abilities were higher than those of the flavonoid extract of ‘Qi-Nan’ extracted by Lin et al. [38], which is presumed to be the result of process optimization, leading to the enhancement of the flavonoid content, which in turn affects its antioxidant capacity. The combination of the two methods suggests that the flavonoid extract of ‘Qi-Nan’ leavefhas a strong antioxidant capacity. 

It can be seen from Figure 10c that the sample and VC concentrations were positively correlated, with the total reducing capacity being in the range of 12.5–200 ug/mL. In the range of 12.5 ug/mL, the total reducing capacity of the flavonoid extract was closer to that of the VC, with no significant difference (*p* > 0.05). When the concentration exceeded 12.5 ug/mL, the total reducing power of the extract and VC gradually showed differences, and the total reducing power of the VC was significantly higher than that of the extract at the same concentration (*p* < 0.05). The reason may be that the difference in the number of available hydrogen atoms from the flavonoid sample was not significant at low concentrations, but as the concentration increased, the flavonoids increased and could provide more hydrogen atoms to participate in the redox reaction, thus promoting the production of Prussian blue, which is similar to the findings of Yin et al. [41].

### 2.5. Acetylcholinesterase-Inhibitory Capacity

**Figure 11:**
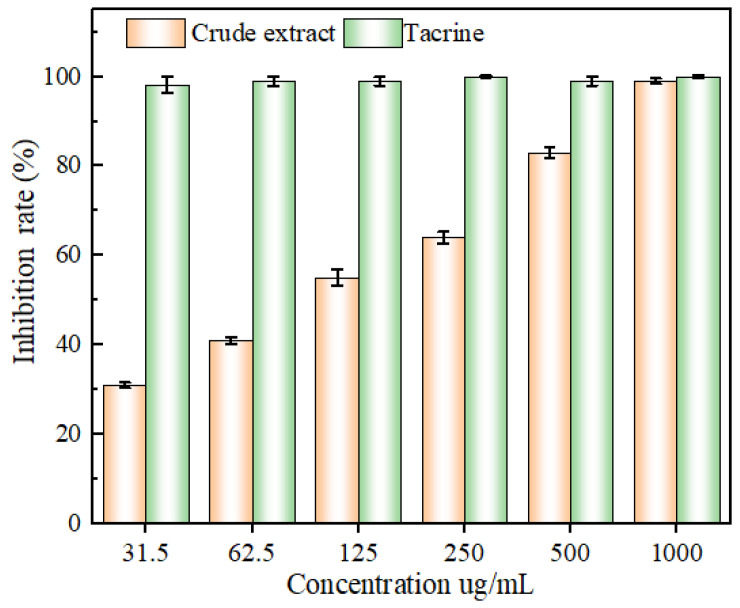
The inhibitory capacity of acetylcholinesterase by mass concentration of flavonoid extract and tacrine.

As illustrated in Figure 11, the inhibition of acetylcholinesterase activity increased and then levelled off, exhibiting an obvious dose dependence. The positive control tacrine inhibited acetylcholinesterase activity better than the extract in the range of 31.3–1000 µg/mL. However, once the concentration of the extract solution was 1000 µg/mL, the inhibition of acetylcholinesterase activity reached 99.18 ± 0.76 %, which was comparable to that of tacrine (100 ± 0.73 %). The IC_50_ values of the extract and tacrine were 102.31 ± 2.43 and 0.08 ± 0.03 μg/mL, respectively, suggesting that the flavonoid extracts of the leaves of ‘Qi-Nan’ possessed the ability to inhibit acetylcholinesterase activity. These demonstrated the inhibitory impact of ‘Qi-Nan’ leaves flavonoids on acetylcholinesterase activity. Additionally, over a certain concentration range, the thatch berry root flavonoid extract’s capacity to inhibit acetylcholinesterase was positively connected with its mass concentration, with an IC50 value of 400.3 μg/mL, which was comparable to the findings of the current investigation.

### 2.6. Glucosidase-Inhibitory Capacity

**Figure 12:**
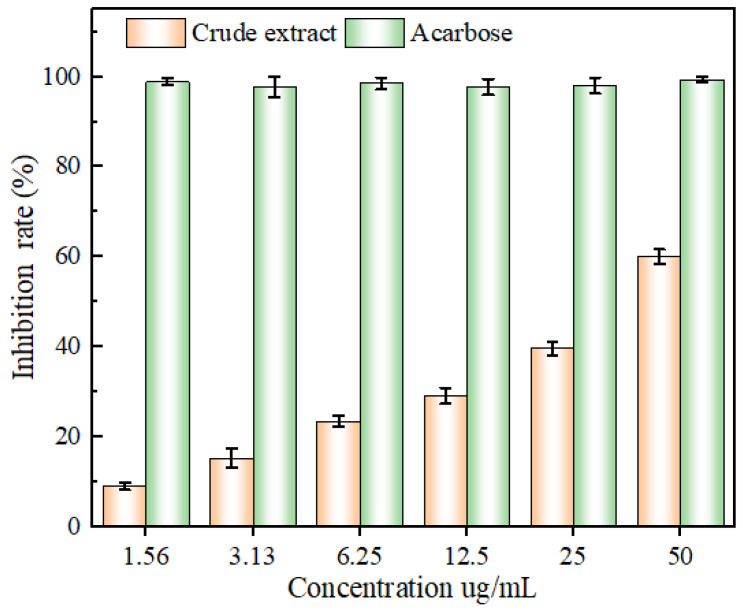
The inhibitory capacity of glucosidase by mass concentration of flavonoid extract and tacrine.

As can be seen in Figure 12, following the increase in the concentration of each sample, the ability to inhibit α-glucosidase activity initially ascended and then stabilized, and all of them showed an obvious quantitative effect relationship. From 1.56 to 50 ug/mL, the inhibitory ability of different concentrations of acarbose on α-glucosidase activity was higher than that of the same concentration of extract. Further, the IC50 values of the extract and acarbose were 38.76 ± 2.34 and 0.05 ± 0. 03 μg/mL, respectively. It is worth confirming that the α-glucosidase inhibitory ability of the ‘Qi-Nan’ flavonoid extracts was superior to that of Mulberry flavonoid extracts, as well as superior to that of Opisthopappus taihangensis flavonoid extracts [42,43]. In another case, the results of this experiment were paralleled by the inhibition of α-glucosidase activity by cornhusk flavonoids [44]. Accordingly, the flavonoids of the leaves of ‘Qi-Nan’ exhibited an excellent α-glucosidase-inhibitory activity, which is valuable for the prevention, as well as the treatment, of diabetes mellitus.

## 3. Materials and Methods

### 3.1. Plant Materials and Chemical Products

In this study, nine-year-old healthy ‘Qi-Nan’ trees without diseases from the Nalou plantation in Nanning City, Guangxi, China (22°60′65″ N, 108°63′29″ E), and their leaves, except for the early ones, were harvested. The leaves were then dried naturally, crushed, and sieved through a 100-mesh sieve.

Anhydrous ethanol, chromatographic-grade methanol, sodium sulfite, aluminum nitrate, sodium hydroxide, rutin standard, 1,1-diphenyl-2-trinitrophenylhydrazine (DPPH^•^), 2,2-benzodiazepine-bis-3-ethylbenzothiazoline-6-sulphonic acid (ABTS^•+^), ascorbic acid (VC), potassium ferricyanide, ferric trichloride, potassium persulphate, acetylcholinesterase, tacrine, α-D-glucosidase, and other experimental drugs (except methanol) were analytically pure.

### 3.2. UHPLC-MS

UHPLC-MS was performed for the preliminary identification of flavonoid compounds in the ‘Qi-Nan’ leaves extract. The crude extract and the purified material with the same mass were weighed and redissolved in chromatographic-grade methanol, respectively, and filtered through a 0.22 μm organic microporous filter membrane.

Mass spectrometry conditions: the column was ACQUITY UHPLCBEHC18 (2.1 mm × 50 mm × 1.7 μm). The mobile phases were 0.1 % formic acid in water (A) and methanol (B). The gradient elution conditions were as follows: 0~15.0 min (5%~80% B); 15.0~18.0 min (80%~100% B); 18.0~18.1 min (100%~5% B); and 18.1~21 min (5% B). Moreover, the flow rate was 0.3 mL/min, the column temperature was 30 °C, and the injection volume was 1 μL.

High-resolution mass spectrometry (HRMS) conditions: ESI ion source, positive and negative ion detection modes, temperature of 300 °C; transfer capillary temperature of 320 °C; sheath gas of 206 kPa; auxiliary gas flow rate of 69 kPa; scanning modes of Full MS and Full MS/dd-MS2, with mass range of 100~1000 Da and resolution of first and second scans of 70000 FWHM (Full-Width Harmonicity) and 70000 FWHM (Full-Width Harmonicity), respectively. The resolutions of the primary and secondary scans were 70,000 FWHM (Full Width at Half Maximum) and 17,500 FWHM, respectively.

The possible molecular formulae of the compounds were determined using Thermo Compound Discoverer 3.2 software, and the flavonoids were identified based on the information on cleavage fragment ions from the secondary mass spectrometry, the retention time of the standards, and a comparison with relevant research.

### 3.3. Determination of Flavonoid Content

#### 3.3.1. Extraction of Flavonoids

The flavonoid content was determined by the sodium nitrite–aluminum nitrate chromogenic method. Following ultrasonic-assisted extraction, 0.5 mL of the sample solution was transferred to a 50 mL volumetric tube fitted with a stopper. Next, 0.5 mL of 5% NaNO_3_ solution was added, followed by 0.5 mL of 10% Al(NO_3_)_3_ solution after 6 min, and so on. Lastly, 4 mL of 4% NaOH solution was added, followed by 15 min, and finally, it was diluted to 25 mL using the corresponding concentration of ethanol solution. In parallel, a blank control consisting of 60% *v*/*v* ethanol solution was used, and the absorbance value was determined with a wavelength of 508 nm.

#### 3.3.2. Establishment of Standard Equations

We weighed 10 mg of rutin standard, configured into 0.2 mg/mL of rutin standard solution with 60% ethanol solution, diluted to a gradient concentration of 0.01–0.05 mg/mL, respectively, with the rutin standard mass concentration as the horizontal coordinate and the absorbance value of the wavelength of 508 nm as the vertical coordinate, to establish a standard curve: y = 11.264 x + 0.004, R^2^ = 0.9998.

#### 3.3.3. Flavonoid Yield

The extracted samples were taken, and the absorbance was measured to calculate the calculated yield of flavonoids according to the following formula:

(1) Flavonoid yield (%)=C/M×V×K×100%

where *C* is the concentration under the corresponding rutin curve; *M* is the sample mass/mg; *V* is the sample volume/mL; and *K* is the dilution factor. 

### 3.4. Single-Factor Experiment

#### 3.4.1. Ethanol Concentration as a Single Variable

After precisely weighing 0.5 g of ‘Qi-Nan’ leaves powder, a 50–90% ethanol solution was added to obtain a material–liquid ratio of 1:40 (g: mL). The extraction process was then carried out with the assistance of ultrasonic technology for 30 min, and the supernatant was extracted by centrifuging the mixture at 6000 rpm/min for 5 min. The colorimetric reaction was then conducted using the extracted supernatant. Ultimately, the flavonoid content was calculated.

#### 3.4.2. Extraction Time as a Single Variable

According to the Section 3.4.1 test, the ethanol concentration was changed to 60%. The ultrasonic extraction time varied between 20 and 60 min, and the rest was controlled under the same condition; afterwards, the flavonoid content was calculated.

#### 3.4.3. Material–Liquid Ratio as a Single Variable

According to the Section 3.4.1 test, the ethanol concentration was changed to 60%. The material–liquid ratio was modified to 1:30–1:60, the rest was kept under uniform conditions, and the flavonoid content was subsequently calculated.

### 3.5. Response Surface Optimization

Using the Box–Behnken experimental design principles, response surface tests were created in Design-Expert 11 software. The ethanol concentration, material–liquid ratio, and ultrasound-assisted extraction time were chosen to be the experimental factors. The ethanol concentration ranged from 50% to 70%, the material–liquid ratio from 1:40 to 1:60, and the ultrasound-assisted extraction time from 20 to 40 min. The flavonoid yield in the leaves of ‘Qi-Nan’ were used as the response value.

### 3.6. Antioxidant Activity and Enzyme-Inhibitory Activity

#### 3.6.1. Antioxidant Activity

According to Ma et al.’s method [45], various quantities of extracts were employed in the diphenylpicrophenylhydrazine (DPPH^•^), 2,2′-Azinobis-(3-ethylbenzthiazoline-6-sulphonate (ABTS^•+^) radical scavenging test. In parallel, VC was used as a positive control.

According to Yin et al.’s method [41], various quantities of extracts were employed in the Ferric ion-reducing antioxidant power (FRAP), and VC was used as a positive control.

#### 3.6.2. Inhibitory Activity of Acetylcholinesterase

A modified version of Ma et al.’s [45] approach was employed. After the sample solutions were prepared using extract, an assay was carried out to determine whether the anti-acetylcholine ester activity was inhibited. Tacrine was used as a positive control. 

#### 3.6.3. Inhibitory Activity of Alpha-Glucosidase

The method of Ma et al. [45] was used with modifications to test the various concentrations of ‘Qi-Nan’ leaves extracts for anti-α-glucosidase activity. A positive control was performed with an acarbose.

## 4. Conclusions

In the current study, the optimum extraction process to extract all the flavonoids of ‘Qi-Nan’ leaves was determined to be a 1:50 material-to-liquid ratio, 60% ethanol concentration, and ultrasound-assisted extraction time of 30 min, resulting in an extraction of 6.68%. It was further demonstrated that Quercetin, Vicenin-2, Cynaroside, Miquelianin, Glycitin, Luteolin, Sakurantein, Hispidulin, Glycitein, and Scrophulein were the major flavonoid components. In addition, it was confirmed that the ‘Qi-Nan’ leaves flavonoids possessed a powerful antioxidant capacity, acetylcholinesterase-inhibiting activity, and α-glucosidase-inhibiting activity, which have the potential to be developed into natural antioxidants, hypoglycemic drugs, and Alzheimer’s disease-preventive drugs. For the next step, we will purify and separate the flavonoids in ‘Qi-Nan’ leaves to further explore the value of ‘Qi-Nan’ leaves.

## Figures and Tables

**Table 1:** UHPLC-MS analyses of flavonoids in the leaves of ‘Qi-Nan’.

NO.	Ion Mode	T_R_/min	Molecular Formula	Theoretical Value	Measured Value	Ion Fragment	Compounds
1	[M−H]^−^	7.100	C_15_H_10_O_7_	302.04265	302.04173	301.03538 271.02481 243.02990 109.02950 95.01385	Quercetin [24]
2	[M+H]^+^	7.167	C_27_H_30_O_15_	594.15847	594.15849	595.16575 379.08102 325.07025 295.05933 121.02856 91.05815	Vicenin-2 [25]
3	[M+H]^+^	8.982	C_21_H_20_O_11_	448.10056	448.10066	449.10784 287.05496 153.01833 135.04385	Cynaroside [26]
4	[M+H]^+^	10.083	C_21_H_18_O_13_	478.07474	478.07497	479.08202 303.04959 257.04404 169.01303 135.04428	Miquelianin [27]
5	[M+H]^+^	10.582	C_22_H_22_O_10_	465.12130	446.12155	447.12857 285.07556 242.05705 124.01576 91.05800	Glycitin [28]
6	[M−H]^−^	11.639	C_15_H_10_O_6_	286.04774	286.04796	285.04046 133.02834 132.02000	Luteolin [29]
7	[M+H]^+^	13.290	C_16_H_14_O_5_	286.08412	286.08415	287.09140 167.03389 147.04414 91.05490	Sakurantein [30]
8	[M−H]^−^	13.573	C_16_H_12_O_6_	300.06339	300.06367	299.05611 284.03250 133.02849	Hispidulin [31]
9	[M+H]^+^	14.495	C_16_H_12_O_5_	284.06847	284.06850	284.06847 242.05736 167.03389 124.01550	Glycitein [28]
10	[M+H]^+^	14.497	C_17_H_14_O_6_	314.07904	314.07898	315.08631 300.06281 272.06754 257.04379 243.06512 167.0230	Scrophulein [32]

**Table 2:** Box–Behnken experimental design and results.

NO.	A Material–Liquid Ratio (%)	B Ethanol Concentration (%)	C Extraction Time (min)	Y Flavonoid Yield (%)
1	50	50	20	6.3377
2	60	60	20	6.3474
3	50	60	20	7.1234
4	50	60	30	6.9875
5	40	60	20	6.2899
6	40	60	40	6.3293
7	50	70	40	6.5365
8	50	60	30	7.4755
9	60	60	40	6.1243
10	40	70	20	6.2640
11	50	60	30	6.8673
12	50	70	20	6.2798
13	60	70	30	6.3128
14	50	60	30	7.0574
15	40	50	30	5.7103
16	60	50	30	5.8337
17	50	50	40	6.0182

**Table 3:** ANOVA analysis of variance table for quadratic model.

Source	Sum of Squares	df	Mean Square	F-Value	*p*-Value	Significant
Model	3.44	9	0.3823	10.07	0.0030	**
A	0.0001	1	0.0020	0.0020	0.9656	
B	0.2787	1	0.2787	7.34	0.00302	**
C	0.0076	1	0.0076	0.2003	0.6680	
AB	0.0014	1	0.0014	00366	0.8537	
AC	0.0172	1	0.0172	0.4540	0.5221	
BC	0.0830	1	0.0830	2.19	0.1828	
A^2^	1.26	1	1.26	33.80	0.0007	**
B^2^	1.1.6	1	1.16	30.67	0.0009	**
C^2^	0.3380	1	0.33880	8.90	0.0204	*
Residual	0.2657	7	0.0380			
Lack of fit	0.0556	3	0.0185	0.3528	0.7907	
Pure error	0.2101	4	0.0525			
Total error	3.71	16				

(*) significant; (**) statistically significant.

**Table 4:** Quadratic model fit data.

R^2^	0.9283
Adjusted R^2^	0.8361
Predicted R^2^	0.6714
Adeq Precision	8.5682

## Data Availability

All data are contained within the article.

## Supplementary Materials

The following supporting information can be downloaded at: https://www.mdpi.com/article/10.3390/molecules29081828/s1, Ion fragmentation maps and cleavage path maps of Compound NO.1–NO.10.
