# Supplementary material for: Optimization of Extraction Process and Analysis of Biological Activity of Flavonoids from Leaves of Cultivated ‘Qi-Nan’ Agarwood
Source: Molecules. 2024 Apr 17;29(8):1828. doi: 10.3390/molecules29081828 (PMC11053590; doi:10.3390/molecules29081828)
Supplement: Supplementary file 1 [file molecules-29-01828-s001.zip › molecules-2937845-supplementary.pdf]

Optimisation of Extraction Process and Analysis of Biological Activity from Leaves of Cultivation 'Qi-Nan' Agarwood

Qingle Li, Penglian Wei, Yingjian Li and Yunlin Fu *

School of Forestry, Guangxi University, Nanning 530011, China; 2109302009@st.gxu.edu.cn (Q.L.); liyingjian@gxu.edu.cn (Y.L.); weipenglian@gxu.edu.cn (P.W.)

***	Correspondence: fuyunlin@gxu.edu.cn; Tel.: 0771-3270881**

Compound NO.1, its quasimolecular ion peak m/z 301.03552[M-H]- has the chemical formula $C_{15}H_{10}O_7$; this parent ion (m/z301.03552) first loses one molecule of CO to produce the fragmentation ion m/z 271.02542 fragmentation ions, followed by the loss of another molecule of CO to produce the fragmentation ion m/z243.03026; this parent ion (m/z 301.03552) undergoes RDA cleavage and loses two molecules of CO to produce the fragmentation ion m/z95.01234; this parent ion (m/z 301.03552) undergoes The parent ion (m/z 301.03552) undergoes RDA cleavage and loses two molecules of CO to generate the fragment ion m/z95.01234; the parent ion (m/z 301.03552) undergoes a simple rupture to generate the fragment ion m/z109.02763. Combined with literature and mass spectrometry library, the compound is presumed to be Quercetin.

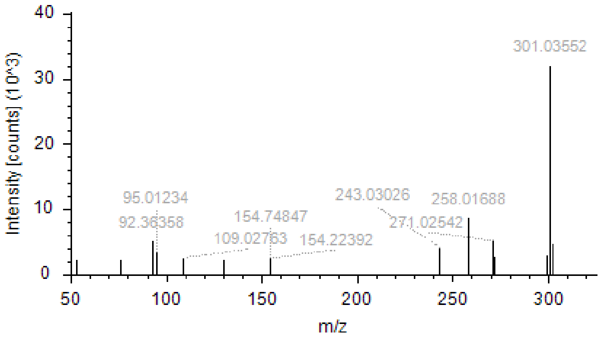

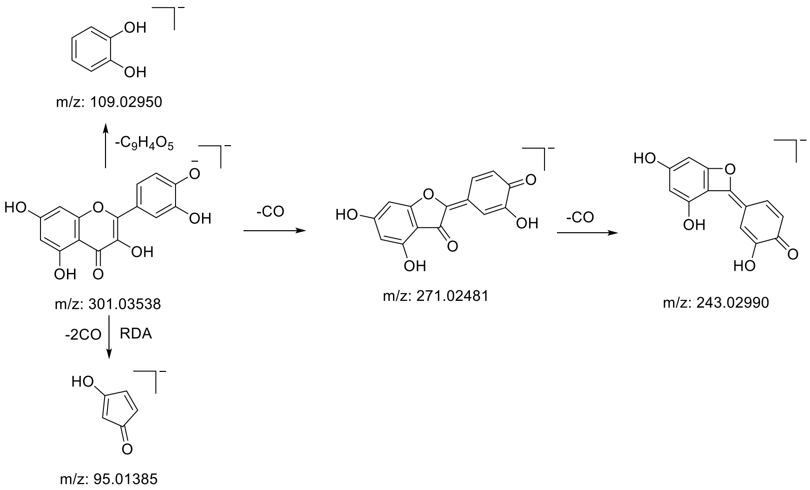

Compound NO.2, Its quasimolecular ion peak m/z595.16575[M+H]+ has the chemical formula $C_{27}H_{30}O_{15}$, and this parent ion (m/z595.16575) firstly dehydrogenates one molecule of sugar and three molecules of water to produce the fragmentation ion m/z379.08102 fragmentation ion, then undergoes RDA cleavage to produce the fragmentation ion m/z325.07025, and finally undergoes retrocyclisation and dehydration to produce the fragment ion m/z91.05815; this parent ion (m/z325.07025) lost one molecule of CO to produce the fragment ion m/z295.05933, followed by reverse cyclisation to produce the fragment ion m/z121.02856, Combined with literature and mass spectrometry library, the compound is presumed to be Vicenin-2.

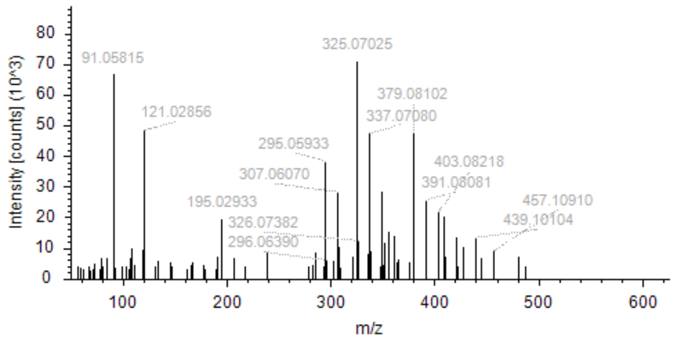

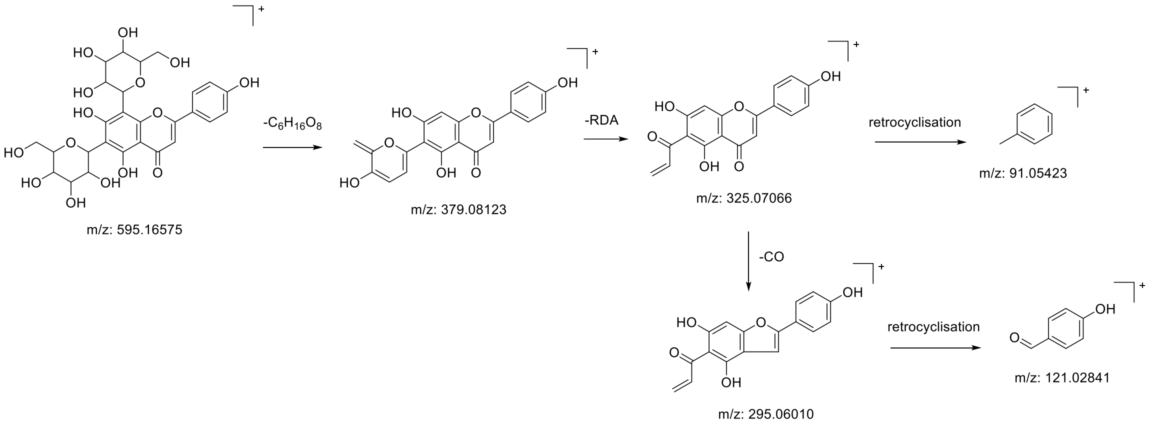

Compound NO.3, Its quasimolecular ion peak m/z449.10784[M+H]+ chemical formula is $C_{21}H_{20}O_{11}$, and the parent ion (m/z449.10784) first loses a molecule of sugar to produce the fragment ion m/z287.05496. The parent ion (m/287.05496) underwent RDA cleavage to form fragment ions m/z153.01833 and m/z135.04385, respectively. Combined with literature and mass spectrometry library, the compound is presumed to be Cynaroside.

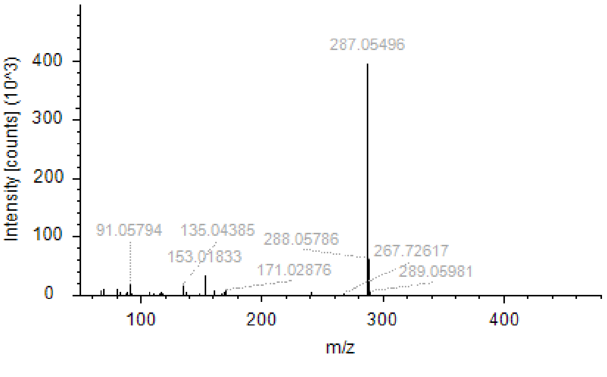

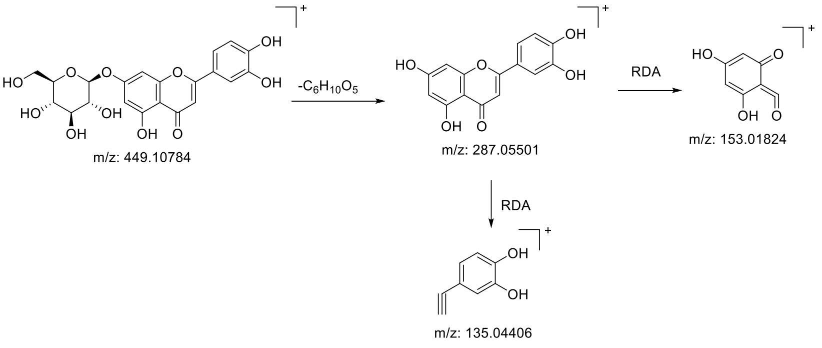

Compound NO.4, Its quasimolecular ion peak m/z479.08202[M+H]+ chemical formula is $C_{21}H_{18}O_{13}$. The parent ion (m/z 479.08202) first loses a molecule of sugar to produce the fragment ion m/z 303.04959 and then loses a molecule of CO to produce the fragment ion m/z257.04404. The parent ion (m/z 303.04959) underwent RDA cleavage to form fragment ions m/z169.01303 and m/z135.04428 respectively. Combined with literature and mass spectrometry library, the compound is presumed to be Miquelianin.

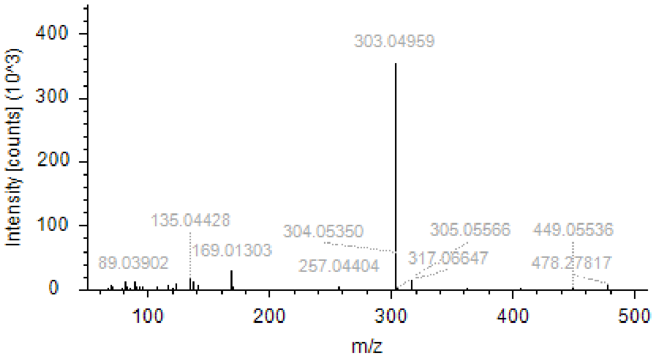

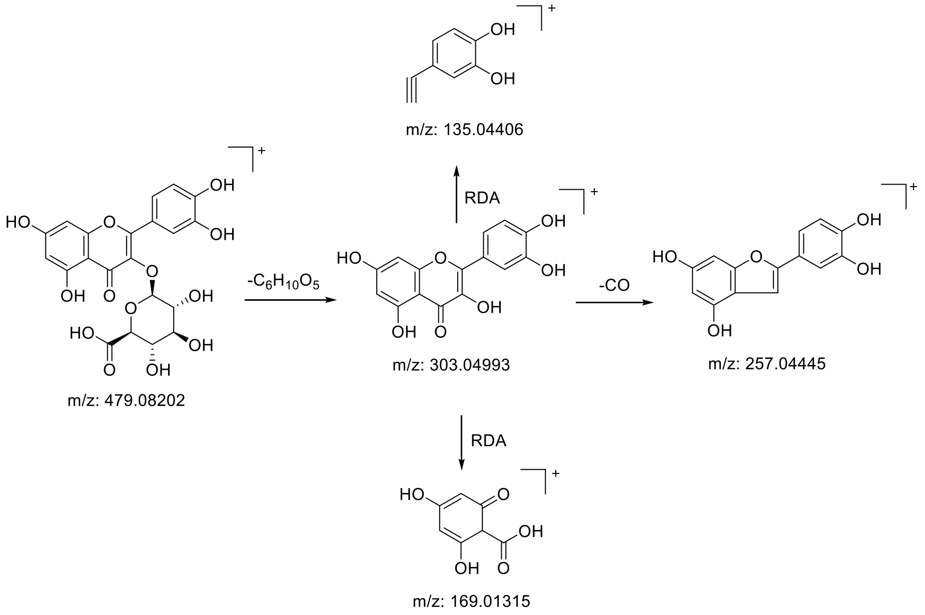

Compound NO.5, Its quasimolecular ion peak m/z447.12857[M+H]+ has the chemical formula $C_{22}H_{22}O_{10}$; this parent ion (m/z 447.12857) first loses a molecule of sugar to produce the fragment ion m/z285.07556 fragment ion, followed by the loss of a molecule of CO to produce the fragment ion m/z242.05705; this parent ion (m/z 285.07556) undergoes RDA cleavage and loses a methyl radical and a molecule of CO to produce the fragment ion m/z124.01576; the parent ion (m/z 285.07556) undergoes a reverse cycloaddition reaction and dehydrates to produce the fragment ion m/z 91.05800. Combined with literature and mass spectrometry library, the compound is presumed to be Glycitin.

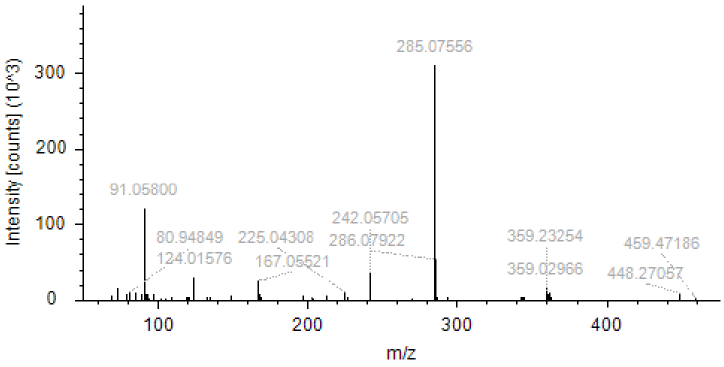

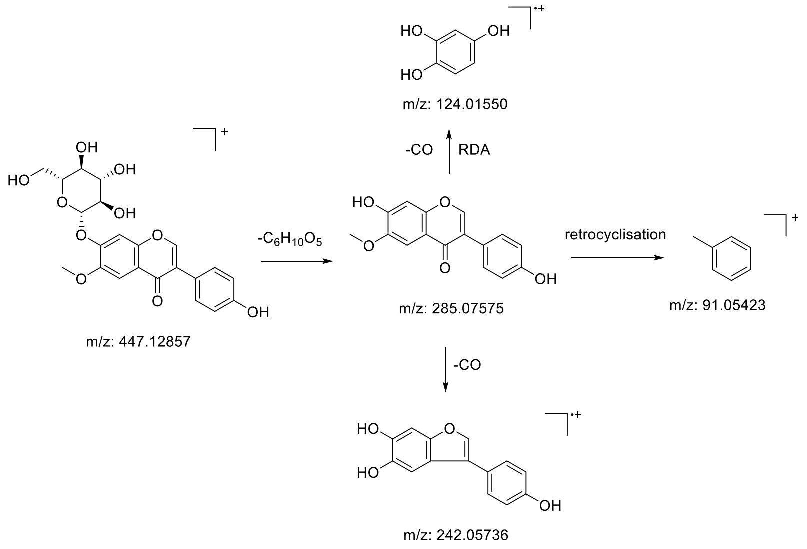

Compound NO.6, Its quasimolecular ion peak m/z 285.04046 [M-H]- the chemical formula is $C_{15}H_{10}O_6$. The parent ion (m/z 285.04046) first underwent RDA cleavage to form fragment ion m/z 133.02834 and then lost hydrogen radical to form fragment ion m/z132.02000. Combined with literature and mass spectrometry library, the compound is presumed to be Luteolin.

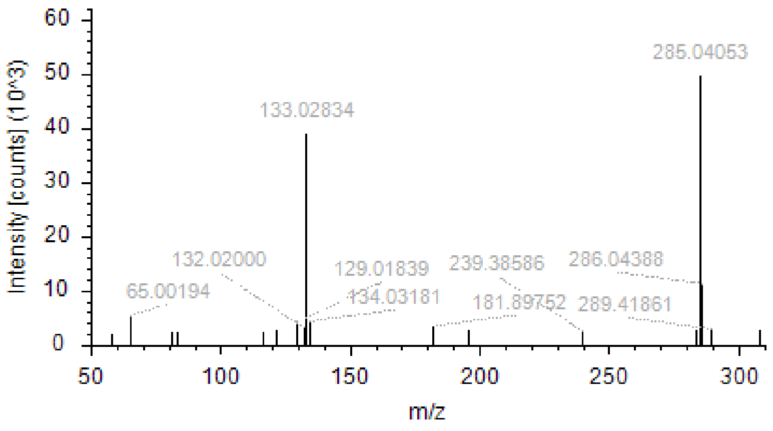

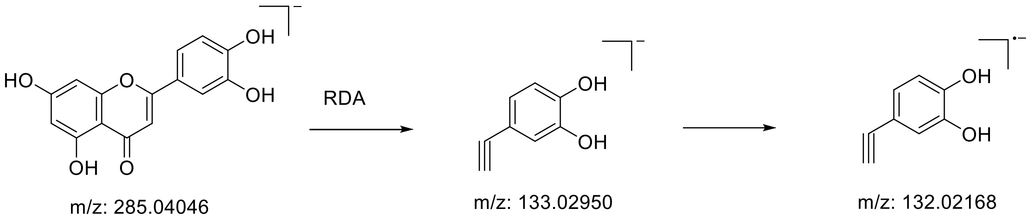

Compound NO.7, Its quasimolecular ion peak m/z287.09140[M+H]+ has the chemical formula $C_{16}H_{14}O_5$, and this parent ion (m/z287.09140) undergoes first a reverse cycloaddition reaction to produce the fragmentation ion m/z147.04414 fragmentation ion, and then a simple fracture to produce the fragmentation ion m/z91.05490; the parent ion (m/z287.09140) undergoes RDA cleavage to produce the fragment ion m/z 167.03389 Combined with literature and mass spectrometry library, the compound is presumed to be Sakuranetin.

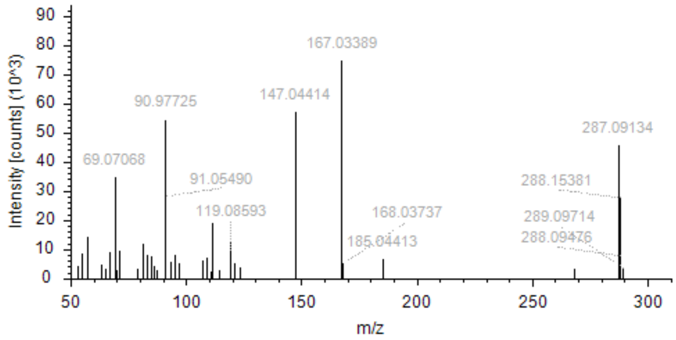

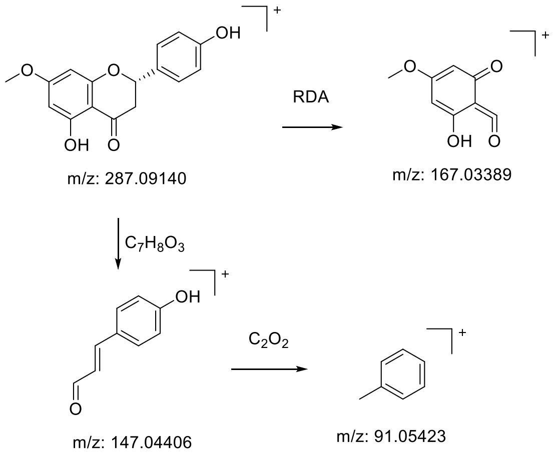

Compound NO.8, Its quasimolecular ion peak m/z 299.05611[M-H]- has the chemical formula $C_{16}H_{12}O_6$, and the parent ion (m/z 299.05611) first loses methyl radicals to produce the fragment ion m/z 284.03250 fragment ions, and then undergoes RDA cleavage and loses a molecule of CO to produce the fragment ion m/z 133.02849. Combined with literature and mass spectrometry library, the compound is presumed to be Hispidulin.

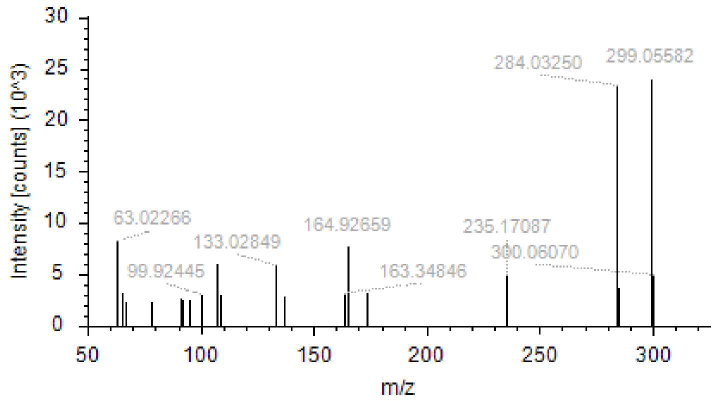

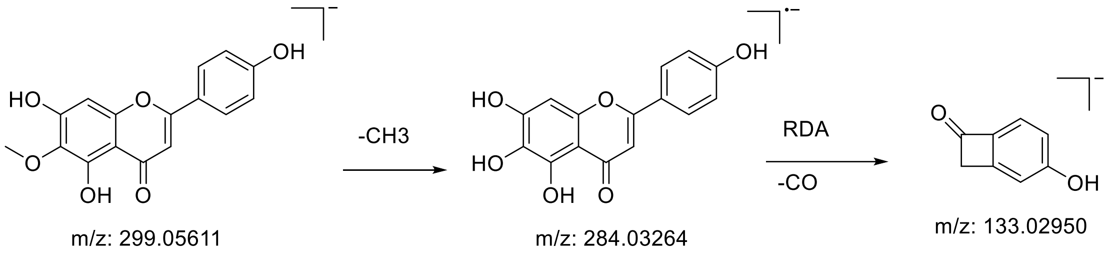

Compound NO.9, Its quasimolecular ion peak m/z284.06847[M+H]+ has the chemical formula $C_{16}H_{12}O_5$, and this parent ion (m/z284.06847) first loses a molecule of CO to produce the fragment ion m/z242.05710 of the fragment ion; the parent ion (m/z284.06847) undergoes RDA cleavage to generate a fragment ion of fragment ion m/z167.03403, which then loses CO and methyl radicals to generate a fragment ion of fragment ion m/z124.01568. Combined with literature and mass spectrometry library, the compound is presumed to be Glycitein.

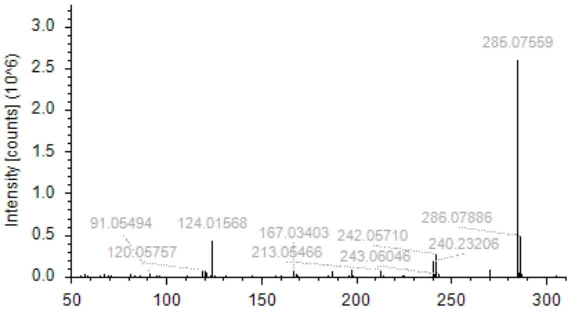

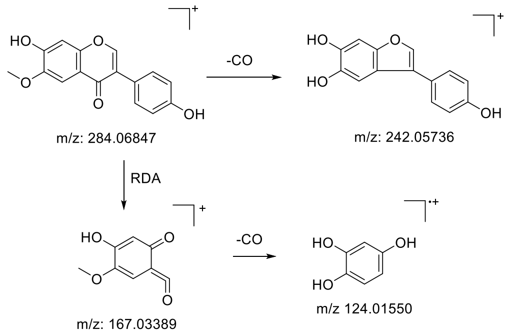

Compound NO.10, Its quasimolecular ion peak m/z315.08631[M+H]+ has the chemical formula $C_{17}H_{14}O_6$, and this parent ion (m/z315.08631) first loses oxymethylene groups to produce the fragmentation ion m/z300.06281 fragmentation ions, then loses a molecule of CO to produce the fragmentation ion m/z272.06754, and loses oxymethyl groups to produce the fragmentation ions m/z 257.04379; This parent ion (m/z 300.06281) undergoes RDA cleavage and dehydrates to produce the fragment ion m/z 167.03355; The parent ion (m/z 300.06281) demethoxylates and loses a molecule of CO to form the fragment ion m/z 243.06512. Combined with literature and mass spectrometry library, the compound is presumed to be Scrophulein.

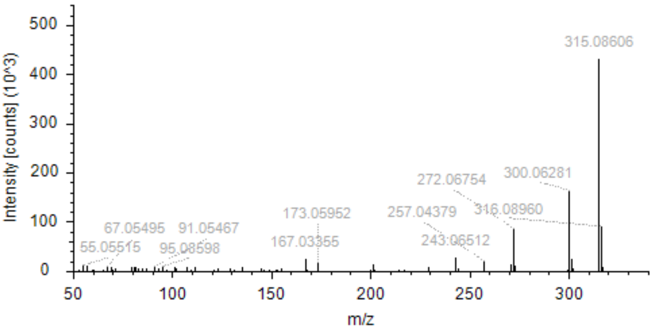
315.08606
300.06281
272.06754
316.08960
257.04379
243.06512
173.05952
167.03355
67.05495
91.05467
55.05515
95.08598
Intensity [counts] (10^3)
m/z

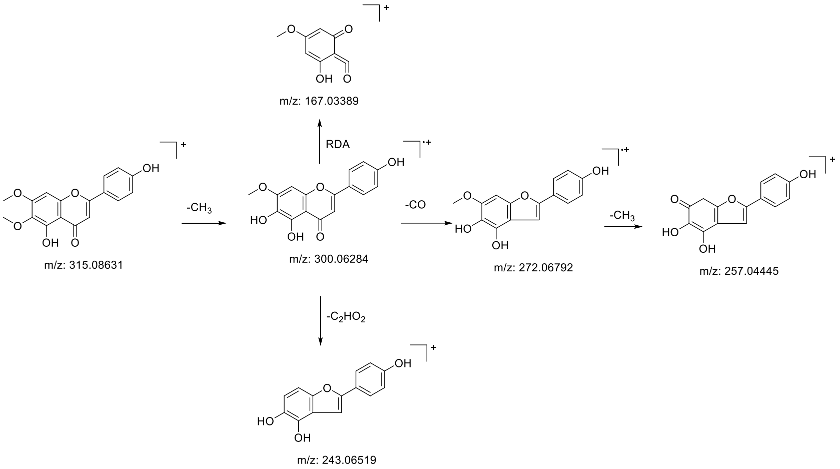
m/z: 315.08631
-CH3
m/z: 300.06284
RDA
m/z: 167.03389
-CO
m/z: 272.06792
-CH3
m/z: 257.04445
-C2HO2
m/z: 243.06519
